# Planning for Your Advance Care Needs (PLAN): A Communication Intervention to Improve Advance Care Planning among Latino Patients with Advanced Cancer

**DOI:** 10.3390/cancers15143623

**Published:** 2023-07-14

**Authors:** Megan J. Shen, Susie Cho, Claudia De Los Santos, Sarah Yarborough, Paul K. Maciejewski, Holly G. Prigerson

**Affiliations:** 1 Division of Clinical Research, Fred Hutchinson Cancer Center, 1100 Fairview Ave. N., Mail Stop D5-290, Seattle, WA 98109, USA; cdelossa@fredhutch.org (C.D.L.S.); syarboro@fredhutch.org (S.Y.); 2 School of Nursing, University of Washington, Seattle, WA 98195, USA; scho2@uw.edu; 3 Department of Radiology, Weill Cornell Medical College, New York, NY 10065, USA; pam2056@med.cornell.edu; 4 Department of Medicine, Weill Cornell Medical College, New York, NY 10065, USA; hgp2001@med.cornell.edu

**Keywords:** cancer, advance care planning, communication, latino

## Abstract

**Simple Summary:**

The goal of this study was to develop an intervention designed to help improve engagement in advance care planning among Latino patients with advanced cancer. Specifically, this study aimed to include feedback from key users of the intervention—Latino patients with advanced cancers, their caregivers, and clinicians and researchers working with this population—to ensure it was optimized to meet the needs of this patient population. Results from this study were used to modify and improve this intervention to be tested in future research for its acceptability, usability, and ability to improve engagement in advance care planning among Latino patients. Future research is needed to demonstrate the intervention effects on reducing clearly documented health disparities in advance care planning completion and end-of-life care received among Latino patients with advanced cancer.

**Abstract:**

Background: The goal of this study was to develop and optimize an intervention designed to address barriers to engagement in advance care planning (ACP) among Latino patients with advanced cancer. The resulting intervention, titled Planning Your Advance Care Needs (PLAN), is grounded in theoretical models of communication competence and sociocultural theory. Materials and Methods: An initial version of the PLAN manual was developed based on a prior intervention, Ca-HELP, that was designed to improve communication around pain among cancer patients. PLAN uses this framework to coach patients on how to plan for and communicate their end-of-life care needs through ACP. In the present study, feedback was obtained from key stakeholders (*n* = 11 patients, *n* = 11 caregivers, *n* = 10 experts) on this preliminary version of the PLAN manual. Participants provided ratings of acceptability and feedback around the intervention content, format, design, modality, and delivery through quantitative survey questions and semi-structured qualitative interviews. Results: Results indicated that the PLAN manual was perceived to be helpful and easy to understand. All stakeholder groups liked the inclusion of explicit communication scripts and guidance for having conversations about ACP with loved ones and doctors. Specific feedback was given to modify PLAN to ensure it was optimized and tailored for Latino patients. Some patients noted reviewing the manual motivated engagement in ACP. Conclusions: Feedback from stakeholders resulted in an optimized, user-centered version of PLAN tailored to Latino patients. Future research will examine the acceptability, feasibility, and potential efficacy of this intervention to improve engagement in ACP.

## 1. Introduction

A critical step for providing quality care for patients with advanced metastatic cancer is to ensure they receive care aligned with their own goals and values [1]. Essential to ensuring patients with serious illnesses receive value-concordant care is their engagement in advance care planning (ACP), which includes having end-of-life (EoL) care conversations and completing advance directives (e.g., living will, health care proxy). ACP is linked with a higher quality of life [2] and medical care [3] at the EoL, such as lower rates of ventilation or resuscitation in the final days of life. Despite the known benefits of ACP, less than half of patients with advanced cancer have conversations to plan for their EoL care [4,5] or complete advance directives [6].

Failure to engage patients with advanced cancer in ACP is higher among Latino patients. Latinos are less likely than non-Latino, Whites (hereafter “Whites”) to engage in ACP [7,8,9,10,11], complete advance directives (9% vs. 67%), name a health care proxy (4% vs. 59%), or have EoL care discussions (32% vs. 85%) [7]. These lower rates of ACP put Latinos at risk for higher rates of suffering and lower quality of care at the EoL. Latinos compared to Whites have higher rates of futile aggressive care (e.g., CPR, ICU stays) at the EoL that does not prolong survival [12,13,14], is often contrary to their preferences and values [15,16,17,18,19,20], is associated with poorer quality of life [4,15,21,22], and is very costly [12].

Despite Latino patients being at the highest risk for ACP disparities of any minority group [8], there are few ACP interventions tailored to meet their ACP needs. Most interventions designed to improve engagement in ACP among Latino patients have focused on targeting older adults [23] but not specifically those with advanced or terminal illnesses. A recent systematic review (in 2021) of ACP, palliative care, and end-of-life care interventions for racial and ethnic underrepresented groups found six studies targeted Latino/Hispanic patients [23]. Of these, the majority were educational. For instance, Cruz-Oliver et al. [24] used educational telenovelas to teach Latino caregivers in community-based settings about end-of-life caregiving. Heyman et al. [25] targeted Latino older adults receiving home care, and the program is a 45-to-60 min session focusing on bilingual ACP/advance directive education. Sudore et al. [26] targeted Latino older adult patients through the “PREPARE for Your Care” online, interactive website and supporting advance directive documents. The only current intervention focused on targeting Latino patients with advanced cancer is Fischer et al. [27], whose prior work focuses on a culturally tailored patient navigator intervention designed to improve education around ACP, advance directives, palliative care, and hospice.

Fischer’s study [27], which is an excellent contribution to the literature on improving ACP among Latino patients with advanced cancer, showed significant improvements in the documentation of ACP. But this intervention was designed to improve palliative care outcomes, not to guide patients through the communication and planning of ACP specifically. As such, it did not significantly shift end-of-life care outcomes such as pain management, hospice use, length of stay, or intensive care at the end-of-life that can be tied to careful planning and communication around ACP. To address this gap in the literature, we designed an intervention designed to target communication around ACP.

In designing an effective intervention for increasing Latinos’ engagement in ACP, it is necessary to consider what contributes to Latinos’ low rates of ACP. First, as highlighted by a narrative review of the literature, a lack of knowledge about advance directives [28] contributes to less engagement in ACP. Second, many Latinos prefer a family-centered approach to determining EoL goals of care [28]. Finally, distinct cultural and religious beliefs about EoL care also reduce interest in engagement in ACP [14,16,17,29,30]. Based on these findings, culturally sensitive communication about ACP that incorporates Latino patients’ beliefs into ACP processes may be a promising approach to boost their engagement in ACP.

Prior research indicates communication around ACP is a promising yet overlooked intervention target to reduce Latino-White disparities in EoL care. Results from one prior study demonstrated that, among Latino patients with advanced cancer who participated in ACP by completing a do not resuscitate (DNR) order or having an EoL care discussion, none received futile aggressive care at the EoL [31]. Results from another study demonstrated that having an EoL discussion with Latino advanced cancer patients improved their likelihood of having their wishes honored with a DNR order tenfold [32].

**Figure 1:**
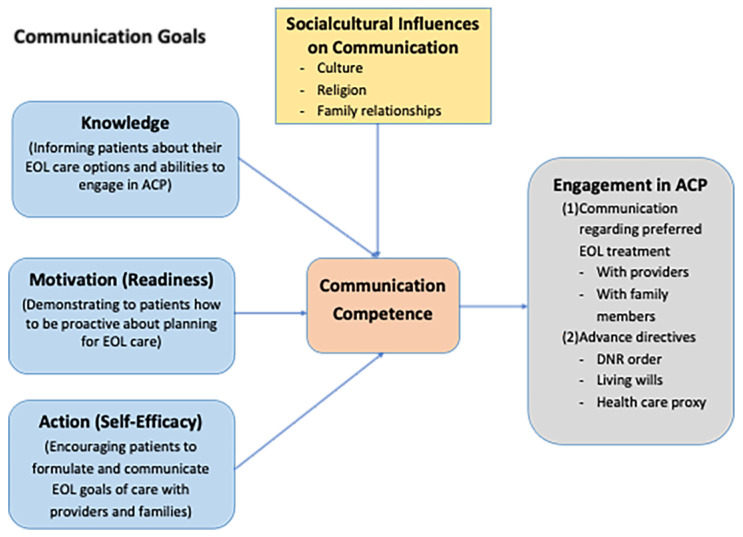
Conceptual model of the role of communication competence and social psychological influences on engagement in ACP among Latino patients. Note: ACP = advance care planning, EOL = end of life.

The goal of the present study was to develop a culturally competent communication coaching intervention to promote ACP among Latino patients with advanced cancer: Planning for Your Advance Care Needs (PLAN). PLAN is grounded in the theoretical models of communication competence [33,34] and sociocultural theory [35] (see Figure 1). More specifically, culturally competent communication [36] refers to communication that focuses on incorporating social and cultural context into communication to reduce racial and ethnic disparities in quality care. Sociocultural theory, which is a social psychological theory, examines how social relationships and culture influence behaviors. Sociocultural theory provides a useful framework for considering how one’s social relationships and cultural beliefs could be integrated into ACP processes and communication to motivate patients to engage in ACP. Combined, these two constructs provide a useful framework for understanding the influence of social relationships and culture on engagement in ACP, which is the focus of the PLAN intervention. In short, PLAN seeks to improve patients’ communication regarding ACP and EoL care through: (1) Knowledge (informing patients of EoL care and ACP options), (2) Motivation (coaching about proactive planning), and (3) Action (training ways to communicate their values with families and providers).

## 2. Methods

### 2.1. Participants and Procedures

The present study used a mixed methods design to obtain feedback on a preliminary version of our developed intervention, PLAN. All study procedures were approved by the Institutional Review Board (IRB) of the participating site. All participants provided informed consent. Participants—which included Latino patients with advanced cancer, their caregivers, and experts (researchers and clinicians) working with this patient population—were recruited from August 2018 to September 2019 from a Northeast academic medical center in an urban setting. Participants were identified through referrals from oncology providers and through medical record chart reviews by study staff. Eligibility criteria for patients included: (1) Age 18 years or older; (2) a diagnosis of locally advanced or metastatic cancer; (3) oncologist-reported prognosis of ≤12 months; (4) physically and cognitively capable of completing informed consent and study procedures as indicated by the treating oncologist (i.e., not too confused or cognitively impaired; not too ill or weak); (5) fluent in English or Spanish and (6) self-identify as Hispanic or Latino. These criteria were selected to identify terminally ill cancer patients. Eligibility criteria for caregivers included the following: (1) age 18 years or older; (2) the person (unpaid, informal caregiver) whom the patient indicates provides most of their informal care; (3) physically and cognitively capable of completing informed consent and study procedures as indicated by the study team member and (4) fluent in English or Spanish. Eligibility criteria for experts included the following: (1) age 21 years or older; (2) having a clinical practice and/or conducting research with advanced cancer patients for 3 years or longer and (3) fluent in English or Spanish.

After consenting to the study, participants were emailed a digital copy or given a hard copy (via mail or in person) of the intervention booklet in their preferred language (English or Spanish). They were then given a week to review study materials. After reviewing the manual, participants engaged in a 45-to-60-minute interview that contained a brief survey and a semi-structured qualitative interview to obtain feedback on the intervention. This interview guide included a brief “Think Aloud” [37] exercise, in which participants interacted with the intervention manual and gave their feedback. The interview guide included questions about content, confusing or difficult parts of the manual, sections that were helpful or unhelpful, preferred added content, thoughts and suggestions for improvement, and specific feedback for each of the five modules of the intervention booklet. The sampling strategy used for this study was a convenience-based sample of patients, caregivers, and experts who met the eligibility criteria of the study.

### 2.2. Intervention: Planning for Your Advance Care Needs (PLAN)

A preliminary version of PLAN was developed based on the following: (1) a prior intervention designed to improve cancer patients’ communication regarding pain management [38,39,40]; (2) a prior model to improve ACP behavior among older adults [41]; (3) our pilot data indicating patients prefer direct communication from oncologists and integration of family members into ACP conversations [42] and (4) input from researchers and oncologists working with this patient population. The PLAN intervention booklet included the following modules [39]: (1) Assessment of current knowledge, motivation, and self-efficacy (action); (2) Clarification and correction of misconceptions about ACP and EoL care; (3) Teaching of relevant concepts; (4) Planning (identifying and creating achievable goals of care and strategies to communicate goals of care to providers and family members) and (5) Rehearsal of communication skills using example communication scripts and role play exercises. The goal was that after working through all five modules, patients would be able to implement the learned ACP communication skills within clinical encounters and/or conversations with family members or loved ones (see Table 1).

### 2.3. Measures

*Sociodemographic and disease characteristics*. Patients and caregivers reported their age, gender (male/female), ethnicity (Hispanic/Latino vs. non-Hispanic/non-Latino), race, country of origin, number of generations the family has been in the U.S., relationship status (married/partnered vs. not married/partnered), employment (employed vs. not employed), education (high school or less, some college, college or more), income (≤$39,999 vs. ≥$40,000), insurance coverage (insured vs. not insured), and whether they spoke Spanish (yes/no). Patients also reported their current cancer stage and caregivers reported their relationship to the patient. Language as a barrier was assessed with an acculturation item asking: “Has language ever been a problem for you while you/your loved one has been receiving care from his/her oncologist?” (yes/no). Experts reported their age, gender, ethnicity, and race. They also reported whether they currently worked with patients with advanced cancer, how many years they had worked with this population, their professional role/background, and frequency of engagement in ACP conversations with patients and their loved ones.

*Engagement in ACP conversations*. This was measured among patients using our previously utilized 8-item measure [42] of discussing EoL care, do-not-resuscitate (DNR) orders, living will, and health care proxy/durable power of attorney with the family and doctor. Caregivers’ engagement in ACP conversations was assessed for EoL care discussions only. Response options included: yes (coded as “1”) and no (coded as “0”).

*Ratings of acceptability*. Patient, caregiver, and expert participants rated the overall perceived helpfulness of the intervention as: “Overall, how helpful do you think PLAN would be for helping you/the patient complete your/the patient’s advance care planning?” (1 = not at all helpful, 5 = very helpful). Participants rated the helpfulness of the questions and interactive materials on the same 5-point Likert scale. Finally, participants rated how difficult it was to understand the content of PLAN (e.g., language, ideas) on a 5-point Likert scale from “not at all” (1) to “5” (very much), with higher ratings indicating more difficulty (e.g., less acceptability).

*Feedback for intervention design, format, and delivery*. Through semi-structured interviews, participants gave feedback on the intervention format and delivery, including font size, colors, number of sessions or modules, frequency of session delivery, location of completing modules, degree to which PLAN related to Latino beliefs when discussing advance care directives, degree to which PLAN would help fill out advance directives and health care proxy forms, preferred modality for delivery (online, mobile device, paper booklet), inclusion of others (e.g., health coach, social worker, family members, etc.), and willingness to complete an online-based intervention.

### 2.4. Statistical Analyses

*Descriptive statistics.* Descriptive statistics were used to examine sociodemographic and health status characteristics, engagement in ACP conversations, and feedback for intervention format and delivery.

*Qualitative analyses.* Qualitative analyses were conducted on the semi-structured interviews to help inform needed modifications to the PLAN manual. All interviews were audio recorded and coded by bi-lingual staff. These coded interviews were then reviewed by trained study team members (MJS, CD) to extract themes. Team member pairs were selected to reflect differing expertise and familiarity with the interviews. They included experts in communication and advance care planning as well as those conducting the interviews who had a familiarity with the study content and interviewing guide. Coded interviews were examined using an inductive thematic text analysis in which conceptual findings emerged through an iterative process of reviewing coded interviews, interpretation, and consensus among coders [43,44,45,46,47]. In Phase 1 of the analysis, team members reviewed each coded interview and noted key findings [48]. In Phase 2, analysis team members transferred key findings into an analysis template categorized by stakeholder type (patient, caregiver, expert, and overall). In Phase 3, team members reviewed coded templates and created a finalized set of codes and extracted themes which were synthesized into a summary of findings that informed the adaptation of the PLAN intervention.

To ensure representative and rigorous data collection was followed, a few actions were taken. First, to ensure data accuracy and reporting, all data and corresponding codes were cross-checked across team members. Second, the targeted number of interviews was pre-determined ahead of time to represent the expected number of participants per subgroup needed to reach data saturation based on our prior studies and prior research, which was *n* = 10 per sub-group (patients, caregivers, and experts). An additional patient (*n* = 1) and caregiver (*n* = 1) were recruited above this originally proposed number to ensure data saturation had been achieved, resulting in a final count of *n* = 11 patients and *n* = 11 caregivers. Data saturation had been achieved at *n* = 10 experts, so no additional experts were recruited. Third, all data excerpts were fully de-identified and anonymized to protect participants’ identities and ensure anonymity. Finally, to enhance the trustworthiness of data, a clear audit trail was kept of the interviewing, recording, coding, and data extraction processes. Additionally, all data were reported back to study team members and members of the Latino community to ensure an accurate representation of study findings.

## 3. Results

### 3.1. Sociodemographic and Disease Characteristics

A total of 11 patients, 11 caregivers, and 10 experts were enrolled in the study. See Table 2 for all patient, caregiver, and expert demographic characteristics. Patients had a mean age of 53.4 (SD = 17.5), and 81.8% (*n* = 9) identified as female. Nearly half (*n* = 5, 45.4%) of patients were considered low-income. All patients (*n* = 11) had insurance and spoke Spanish. Caregivers had a mean age of 44.5 years (SD = 16.9) and 90.9% (*n* = 10) identified as female. The most common type of relationship with the patient reported was a son or daughter (*n* = 4, 36.3%), followed by a spouse or partner (*n* = 3, 27.3%), or friend (*n* = 2, 18.2%). All caregivers (*n* = 11) spoke Spanish. Most patients (*n* = 10, 90.9%) and caregivers (*n* = 9, 81.8%) indicated language was not a problem in communicating with the patient’s oncologist. Experts had a mean age of 44.6 years (SD = 9.7), and 80.0% (*n* = 8) identified as female. Nearly all experts (*n* = 9, 90.0%) were currently working with advanced cancer patients, and half (*n* = 5, 50.0%) had worked with them for 10+ years. The majority (*n* = 7, 70.0%) had ACP conversations “very often.”

### 3.2. Engagement in ACP Conversations

Most patients reported not having had EoL care conversations with their oncologists (*n* = 9, 81.8%), nor had they discussed DNR orders (*n* = 10, 90.9%) or living will (*n* = 8, 72.7%). Most patients (*n* = 8, 72.7%) had discussed health care proxy with their oncologist. With their family, a slight majority of patients reported having discussed EoL care (*n* = 6, 54.5%) and health care proxy (*n* = 6, 54.5%). However, most had not discussed DNR orders (*n* = 8, 72.7%) or living will (*n* = 10, 90.9%). Most caregivers (*n* = 10, 90.9%) had not had an EoL care conversation with the patient’s oncologist but nearly half (*n* = 5, 45.4%) had discussed it with the patient themselves.

### 3.3. Intervention Acceptability

**Figure 2:**
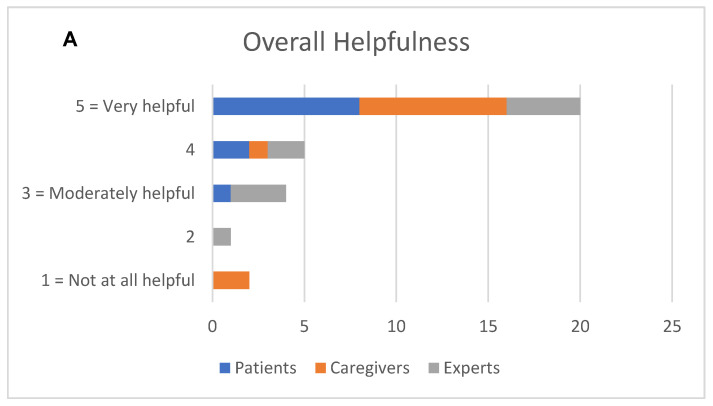

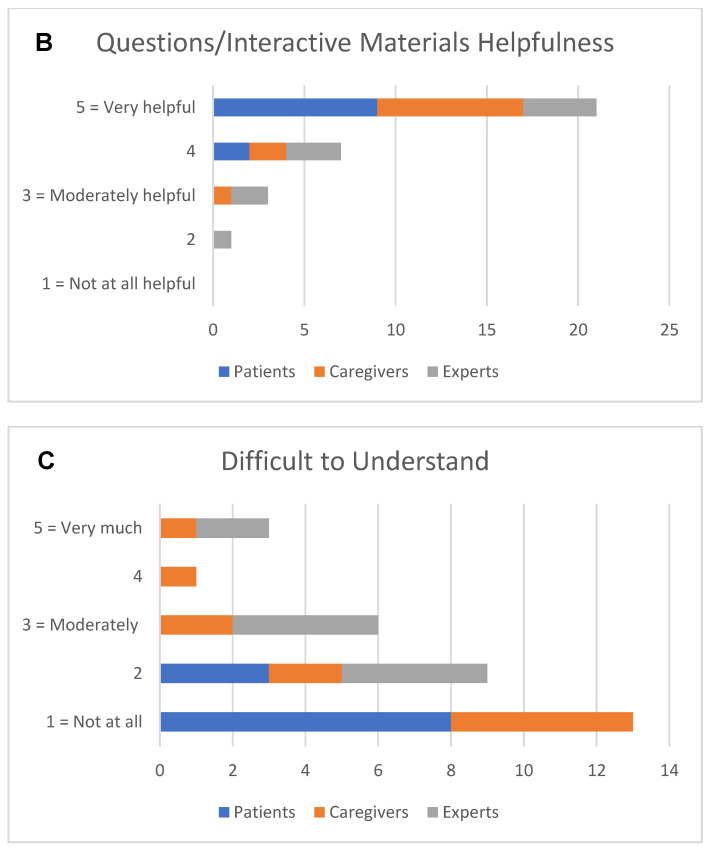
Acceptability ratings among patients (*n* = 11), caregivers (*n* = 11), and experts (*n* = 10) for overall helpfulness of the intervention (**A**), helpfulness of the questions and interactive materials (**B**), and degree of difficutly to understand the intervention (**C**).

Most participants rated the helpfulness of PLAN (to understand and complete ACP), the questions, and the interactive materials highly (see Figure 2A,B). Most participants rated the content of PLAN as “not at all” difficult to understand (see Figure 2C).

### 3.4. Feedback for Intervention Design, Format, and Delivery

Most participants liked the font size, indicating it was readable (*n* = 11 patients, *n* = 10 caregivers, and *n* = 7 experts). Nearly all patients (*n* = 10, 90.9%) and caregivers (*n* = 8, 72.7%) indicated five modules was an acceptable number of sessions whereas half (*n* = 5, 50.0%) of experts indicated it was too many sessions. Over half of the patients (*n* = 6, 54.5%) preferred sessions to be completed once per day whereas caregivers and experts were mixed. Caregivers reported sessions should be completed as one per day (*n* = 2, 18.2%), a couple per week (*n* = 3, 27.3%), one per week (*n* = 3, 27.3%), and all in one day (*n* = 3, 27.3%). Half of the experts (*n* = 5, 50.0%) indicated modules should be completed as a couple per week. Over half of the patients (*n* = 6, 54.5%) and nearly half (*n* = 5, 45.5%) of caregivers preferred completing the intervention at home. However, only *n* = 1 expert (10.0%) reported patients should complete the PLAN manual at home (patients’ preferred location), whereas *n* = 3 (30.0%) reported at the doctor’s office, *n* = 1 (10.0%) at the hospital, and *n* = 5 (50.0%) as “other”.

Most patients (*n* = 7, 63.6%) reported that PLAN was related to Latinos’ beliefs when discussing advance care directives. However, all Latinos who indicated that Latino beliefs were not well-incorporated (*n* = 4, 36.4%) were Spanish-speaking, indicating a gap in content. A total of 36.4% (*n* = 4) of caregivers indicated that PLAN related to Latinos’ beliefs when discussing advance care directives, 36.4% (*n* = 4) indicated it did not, and *n* = 3 (27.3%) indicated they were unsure. Finally, most experts (*n* = 6, 60.0%) indicated they were unsure if PLAN related to Latinos’ beliefs when discussing advance care directives. Recommendations included adding more explicit Latino narratives, stories, and examples, which was integrated into the feedback and modifications.

Overwhelming majorities of patients (*n* = 9, 81.8%) and caregivers (*n* = 10, 90.9%) reported preferring a paper booklet version of the manual. All patients wanted to complete the PLAN manual with someone, including family members (*n* = 5, 45.5%), in a group with cancer patients (*n* = 4, 36.4%), their social worker (*n* = 1, 9.1%), and a health coach (*n* = 1, 9.1%). Similarly, all caregivers wanted their loved one to complete the PLAN manual with someone, including family members (*n* = 5, 45.5%), a social worker (*n* = 3, 27.3%), a peer (*n* = 2, 18.2%), a health coach (*n* = 1, 9.1%), or a group of other cancer patients (*n* = 1, 9.1%). Most experts also reported wanting patients to complete the PLAN manual with someone, including a social worker (*n* = 3, 30.0%), health coach (*n* = 2, 20.0%), or peer (*n* = 1, 10.0%). A total of *n* = 2 (20.0%) indicated “other” and *n* = 2 (20.0%) indicated “don’t know.” If offered online, most patients (*n* = 9, 81.8%) and caregivers (*n* = 7, 63.6%) said they would be willing to try PLAN and most experts (*n* = 6, 63.0%) indicated their patients would be willing to try PLAN.

### 3.5. Qualitative Feedback for Modifications

Overall themes emerged around the strengths, weaknesses, and behavioral activation of PLAN (see Table 3). For strengths, patients, caregivers, and experts overwhelmingly preferred content around how to communicate ACP. Participants liked having scripts for how to communicate about ACP with doctors and loved ones and indicated specific content on communicating with family members was the most helpful. Of note, one patient (P102) indicated having a module devoted to how to talk to your doctor about ACP, which included information about doctors wanting to have these conversations, addressed common beliefs that the “doctor knows best” by indicating that these conversations help the doctor learn valuable information. One caregiver (CG201) indicated that content on integrating family members into ACP would be “the most helpful in making advance care plans”. Experts noted clear explanations of ACP and advance directives were important for their cancer patients.

For weaknesses, a few themes emerged. First, all participants stated that specific terms (e.g., DNR orders, ACP, palliative care) were too technical and confusing. Patients and caregivers also noted that checklists asking about ACP knowledge were confusing and overwhelming (Module 2). A second theme to emerge was the length of the intervention. Patients noted it was too long (originally 36 pages) and hard to follow while patients and caregivers both indicated the manual was repetitive throughout. Experts indicated the language should be more direct (E305), readable at a lower reading grade level (E306), and more visually based. A third theme, noted by patients, was the lack of integration of family members into ACP processes. For key behavioral activation from the intervention, patients noted the intervention encouraged them to have conversations with their oncologist and prompted them to complete or update their advance directives.

**Figure 3:**
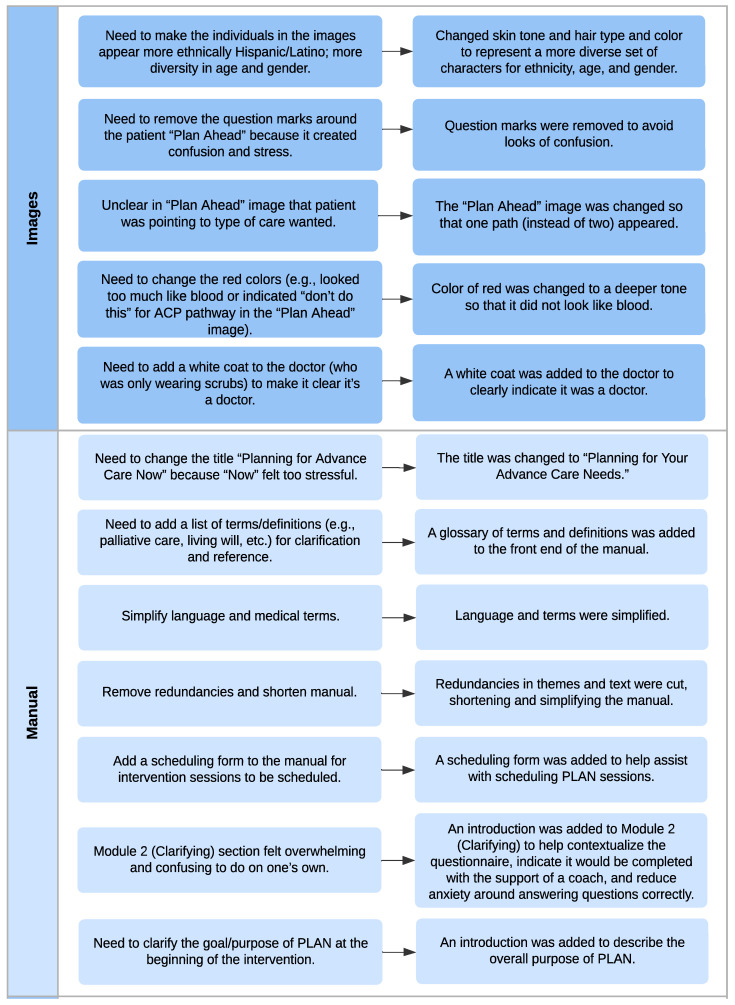

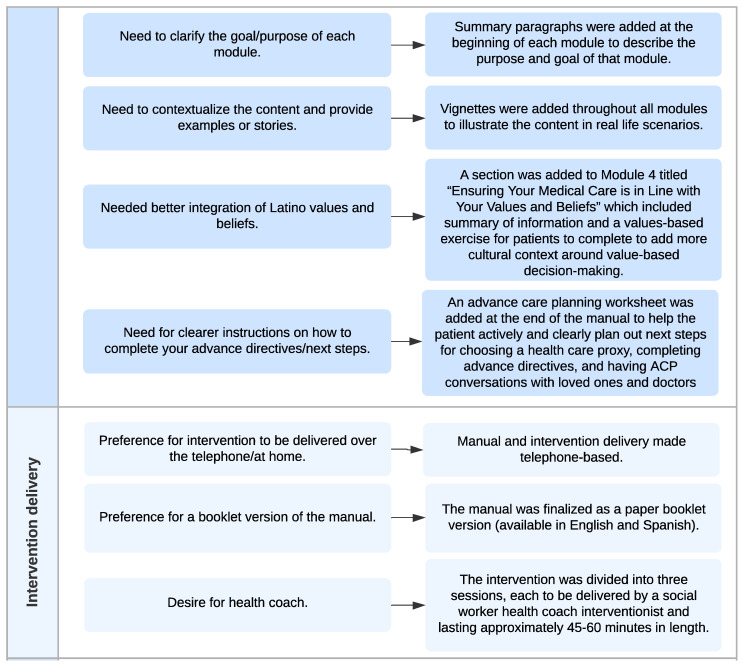
Qualitative feedback on recommended modifications to the PLAN intervention.

Participants also gave feedback around needed modifications to the PLAN manual’s core images (“Plan Ahead” and “Collective Decision Making”), the manual content, and the intervention delivery. This feedback is summarized in Figure 3. In addition to providing feedback for necessary modifications to the images, there were noted strengths. The term “plan ahead” was viewed favorably and prompted action among patients and caregivers. The concept of making decisions together was noteworthy and appreciated among patients and caregivers. Based on the collective feedback, several changes were made to the PLAN manual (Figure 3).

## 4. Discussion

Consistent with prior research [42], patients in the present study had low rates of engagement in ACP and represent a high-need patient population. Results from this study indicate that patients and caregivers found PLAN to be highly acceptable and easy to understand. Perhaps most noteworthy, some patients indicated that reviewing the manual prompted them to either engage in ACP or go back to their current advance directives and make updates. In addition, patients and caregivers rated the manual itself as well as the questions and interactive materials as being very helpful. Experts rated it favorably as well, however, noting it was a bit too difficult to understand and that intervention materials needed to be simplified. Most participants liked the font size, colors, and number of modules. A paper booklet was the preferred modality, and most patients wanted to complete the intervention at home with someone guiding them and involving their family in the process.

The main themes to emerge from the feedback from patients, caregivers, and experts that drove the improvements and modifications to the intervention included the following: (1) the material was too complex and terms were too unfamiliar and needed to be simplified and explained more clearly; (2) there was a clear need to include narratives and real-world examples of the study concepts being applied to culturally sensitive scenarios and situations; (3) there needed to be a clearer integration of values and cultural norms around ACP to the manual; (4) users wanted clear step-by-step action items for carrying out and engaging in ACP and completing advance directives and (5) instructing patients and caregivers in how to communicate about the difficult topic of ACP was valuable and favorable among patients and caregivers.

Major changes were made to the manual based on user feedback and included: making it easier to understand, providing supportive tables and reference materials to understand the medical jargon, simplifying the language and reducing redundancies in content, adding vignettes to give examples of the content in real-world scenarios and make it more applicable to Latinos, adding features around cultural tailoring and inclusion of family members, and providing clear end goal action steps for ACP at the end of the manual. Overall, the resulting revised version of PLAN is designed to walk patients through ACP processes, including having conversations with their families and doctors and receiving step-by-step guidance from a health coach. Future research should include an examination of the potential efficacy of this intervention to improve engagement in advance care planning as well as examine how this intervention might work better or worse among various subgroups of patient populations (e.g., subgroups of Latinos, different cancer sites/types, preferred language).

A significant strength of this study is, of course, the ethnic representation of an underserved patient population who suffers from lower rates of advance directive completion and EoL care conversations [7,8,9,10,11]. This study highlights a much-needed, culturally tailored intervention to begin addressing these major disparities in ACP and the likely quality of EoL care. Given preliminary findings in this study, PLAN holds promise as a potentially acceptable, feasible, and effective intervention to improve rates of engagement in ACP among Latino patients and caregivers, resulting in a reduction of ethnic disparities in ACP.

Despite these strengths, there are limitations that must be acknowledged in interpreting results. First, although some heterogeneity in Latino subgroup type exists (e.g., Puerto Rican) in the present sample, there are not enough data to determine if major feedback or themes differed by these groups. If found to be an effective intervention in future research, PLAN could be further tailored to target specific Latino subgroups. Second, the sample size although enough to reach thematic saturation, is small and was not able to represent variations across age, gender, or other sociodemographic characteristics. As such, it is possible certain components or themes of the manual will resonate better with certain patient subgroups than others. Third, this study was conducted at a major academic medical center in an urban setting, which may limit the degree to which this manual can be tailored for broader populations.

### Recommendation for Practice

These study findings indicate that our developed intervention PLAN, which represents a culturally tailored intervention designed to improve engagement in ACP among Latino patients with advanced cancer, is acceptable among key stakeholders which is critical for uptake and clinical integration. PLAN shows promise for improving engagement in ACP among this patient population. Future research is needed to determine the feasibility, acceptability, and potential efficacy of PLAN. If future research indicates PLAN is feasible, acceptable, and has efficacy, PLAN could represent an effective intervention that can be integrated within clinical practice to improve ACP among this vulnerable population of Latino patients with advanced cancer.

## 5. Conclusions

Modifications made to the PLAN manual reflect expressed needs of this patient population around ACP, including increased clarity and knowledge about advanced directives [28], a family-centered approach to determining EoL goals of care [28], and integration of cultural and religious beliefs in this planning process [14,16,17,29,30]. The intended goal of the PLAN manual, which was co-created with stakeholder feedback, is to address the gap in the literature on the need for a culturally competent communication intervention [36] designed to improve engagement in ACP among Latino patients. Future research will examine the feasibility, acceptability, fidelity, and potential efficacy of PLAN to improve rates of engagement in ACP among Latino patients with advanced cancer.

## Figures and Tables

**Table 1:** PLAN modules (table of contents appearing in the workbook/intervention manual).

Module	Content
Module 1: Checking Awareness	What is Advance Care Planning and How is it Relevant to Me and My Family and Loved Ones?
Module 2: Clarifying	Common Misconceptions about Advance Care Planning and Treatment for Seriously Ill Patients
Module 3: Knowledge Transferring	Understanding Advance Care Planning and Treatment Options for Seriously Ill Patients
Module 4: Goal Setting	Identifying Your Goals of Care and Planning How to Make Them Known and Carried Out
Module 5: Rehearsing	How to Talk to Your Family, Loved Ones, and Doctors about the Type of Care You May Want: Communicating Advance Care Plans

**Table 2:** Sociodemographic, disease, and practice characteristics for patients (*n* = 11), caregivers (*n* = 11), and experts (*n* = 10).

Sociodemographic and Clinical Characteristics			
	Patients (N = 11)	Caregivers (N = 11)	Experts (N = 10)
	Mean (SD)		
Age (years) *	53.4 (17.5)	44.5 (16.9)	44.6 (9.7)
**Gender**	**N (%)**		
Male	2 (18.2%)	1 (9.1%)	2 (20.0%)
Female	9 (81.8%)	10 (90.9%)	8 (80.0%)
**Hispanic/Latino**			
Yes	10 (90.9%)	11 (100.0%)	3 (30.0%)
No	0 (0.0%)	0 (0.0%)	7 (70.0%)
Missing	1 (9.1%)	0 (0.0%)	0 (0.0%)
**Race**			
White or Caucasian	3 (27.3%)	3 (27.3%)	7 (70.0%) 3 (30.0%)
Black or African American	1 (9.1%)	0 (0.0%)	
Asian	0 (0.0%)	0 (0.0%)	
American Indian or Alaskan Native	0 (0.0%)	0 (0.0%)	
Native Hawaiian or Other Pacific Islander	0 (0.0%)	0 (0.0%)	
Multi-Racial	0 (0.0%)	0 (0.0%)	
Other (Hispanic or Latino)	7 (63.6%)	7 (63.6%)	
Other	0 (0.0%)	1 (9.1%)	
**Country of Origin**			
African Country	1 (9.1%)	0 (0.0%)	-
Cuba	1 (9.1%)	0 (0.0%)	
Dominican Republic	3 (27.3%)	6 (54.5%)	
Ecuador	1 (9.1%)	1 (9.1%)	
Honduras	0 (0.0%)	1 (9.1%)	
Mexico	1 (9.1%)	0 (0.0%)	
Puerto Rico	4 (36.3%)	3 (27.3%)	
**Number of Generations Family Has Been** **in US**			
First to move to US	5 (45.4%)	7 (63.6%)	-
1	3 (27.3%)	1 (9.1%)	
2	1 (9.1%)	2 (18.2%)	
3 or more	2 (18.2%)	1 (9.1%)	
**Relationship Status**			
Married/partnered	3 (27.3%)	6 (54.5%)	-
Not Married/partnered	8 (72.7%)	5 (45.4%)	
**Employment Status**			
Employed	1 (9.1%)	6 (54.5%)	-
Not employed	10 (90.9%)	5 (45.4%)	
**Highest Education Level Completed**			
High school or less	6 (54.5%)	5 (45.4%)	-
Some college	2 (18.2%)	5 (45.4%)	
College or more	3 (27.3%)	1 (9.1%)	
**Total Household Income (Annual)**			
Less than or equal to $39,999	5 (45.4%)	6 (54.5%)	-
Greater than or equal to $40,000	3 (27.3%)	4 (36.3%)	
Refused/don’t know	3 (27.3%)	1 (9.1%)	
**Insurance Status**			
Insured	11 (100.0%)	-	-
Not insured	0 (0.0%)		
**Speaks Spanish?**			
Yes	11 (100.0%)	11 (100.0%)	-
No	0 (0.0%)	0 (0.0%)	
**Caregiver Relationship to Patient**			
Spouse/partner	-	3 (27.3%)	-
Sibling		1 (9.1%)	
Parent		1 (9.1%)	
Son or daughter		4 (36.3%)	
Friend		2 (18.2%)	
**Disease Characteristics**			
**Current Cancer Stage (self-reported)**			
Early stage (Stage I)	0 (0.0%)	-	-
Middle stage (Stage II)	0 (0.0%)		
Late stage (Stage III)	3 (27.3%)		
End stage (Stage IV)	4 (36.3%)		
Don’t know	4 (36.3%)		
**Clinical Practice Questions**			
**Currently Working with Patients with** **Advanced Cancer?**			
Yes	-	-	9 (90.0%)
No			1 (10.0%)
**Years Worked with Patient Population**			
Less than 5 years	-	-	2 (20.0%)
5 years—less than 10 years			3 (30.0%)
10+ years			5 (50.0%)
**Profession**			
Oncologist	-	-	1 (10.0%)
Nurse			2 (20.0%)
Psychologist			1 (10.0%)
Psychiatrist			1 (10.0%)
Other			5 (50.0%)
**Frequency of Engagement in ACP** **Conversations**			
Never	-	-	0 (0.0%)
Rarely			0 (0.0%)
Sometimes			0 (0.0%)
Very often			7 (70.0%)
Always			3 (30.0%)

* Patients’ mean age is only calculated for N = 9 patients, due to missing data. Clinical characteristics were assessed at time of interview and are representative of a patient’s health status at that time.

**Table 3:** Qualitative feedback on the PLAN intervention.

Overarching Theme	Feedback	Stakeholder (s)
Strengths	Content related to how to communicate about ACP/Module 5 (Rehearsing) was a strength	Patients, caregivers, experts
	Liked having scripts for how to communicate with doctors and loved ones	Patients, experts
	Having clear explanations of ACP and advance directives is important for cancer patients	Experts
	Section on communication with family members was the most helpful	Caregivers
Weaknesses	Question checklist in Module 2 (Clarifying) was confusing and overwhelming	Patients, caregivers
	Manual was too long, hard to follow	Patients
	Material repetitive/could be cut down	Patients, caregivers
	Terms throughout manual (ACP, DNR, palliative care) were too technical/confusing	Patients, caregivers, experts
	Need to make language more direct/specific to patient	Experts
	Need to reduce reading grade level	Experts
	Simplify communication of information via visual-based and inclusion of vignettes and stories	Experts
	Need to integrate family members into the ACP process	Patients
	Better integration of Latino narratives and culture into intervention manual	Patients, caregivers
Key takeaways and behavioral activation from PLAN	Including information on doctors wanting to have ACP conversations addressed common belief that “doctors know best” by highlighting these conversations can help the doctor learn valuable information	Patients
	Using this manual prompted completion of advance directives or updating current advance directives	Patients

## Data Availability

Data can be shared and made available upon reasonable request.
